# CDX2-Suppressed Colorectal Cancers Possess Potentially Targetable Alterations in Receptor Tyrosine Kinases and Other Colorectal-Cancer-Associated Pathways

**DOI:** 10.3390/diseases12100234

**Published:** 2024-10-01

**Authors:** Ioannis A. Voutsadakis

**Affiliations:** 1 Algoma District Cancer Program, Sault Area Hospital, Sault Ste. Marie, ON P6B 0A8, Canada; ivoutsadakis@yahoo.com or ivoutsadakis@nosm.ca; 2 Section of Internal Medicine, Division of Clinical Sciences, Northern Ontario School of Medicine, Sudbury, ON P3E 2C6, Canada

**Keywords:** transcription factors, gastrointestinal cancer, genomic classification, microsatellite instability, chromosome instability

## Abstract

Background: Colorectal cancer, a prevalent gastrointestinal carcinoma, has a high risk for recurrence when locally advanced and remains lethal when in an advanced stage. Prognostic biomarkers may help in better delineating the aggressiveness of this disease in individual patients and help to tailor appropriate therapies. CDX2, a transcription factor of gastrointestinal differentiation, has been proposed as a biomarker for good outcomes and could also be a marker of specific sub-types amenable to targeted therapies. Methods: Colorectal cancers from The Cancer Genome Atlas (TCGA) colorectal cohort and colon cancers from the Sidra-LUMC AC-ICAM cohort were categorized according to their expressions of CDX2 mRNA. Groups with CDX2 suppression were compared with cancers showing no suppression regarding their clinical and genomic characteristics. Results: CDX2-suppressed colorectal cancers showed a high prevalence of Microsatellite Instability (MSI) and a lower prevalence of chromosomal Instability (CIN) compared to non-CDX2-suppressed cancers. In addition, CDX2-suppressed cancers had a higher prevalence of mutations in several receptor tyrosine kinase genes, including *EGFR*, *ERBB3*, *ERBB4*, *RET*, and *ROS1*. In contrast, CDX2-suppressed cancers displayed lower mutation frequencies than non-CDX2-suppressed cancers in the genes encoding for the two most frequently mutated tumor suppressors, *APC* and *TP53*, and the most frequently mutated colorectal cancer oncogene, *KRAS*. However, CDX2-suppressed colorectal cancers had a higher prevalence of mutations in alternative genes of the WNT/APC/β-catenin and KRAS/BRAF/MEK pathways. In addition, they showed frequent mutations in DNA damage response (DDR) genes, such as *BRCA2* and *ATM*. Conclusion: CDX2-suppressed colorectal cancers constitute a genomically distinct subset of colon and rectal cancers that have a lower prevalence of *KRAS*, *APC*, and *TP53* mutations, but a high prevalence of mutations in less commonly mutated colorectal cancer genes. These alterations could serve as targets for personalized therapeutics in this subset.

## 1. Introduction

Colorectal cancer, the most frequent gastrointestinal neoplasm, is curable when diagnosed in its early stages. However, when advanced, it remains a significant cause of cancer-related mortality, which has become a focus of public health concerns due to the increasing incidence of this disease [1]. Although the cause of this increasing colorectal cancer incidence has not been entirely clarified, progress in the survival rates of advanced colorectal cancer has been achieved in recent decades with improvements in systemic therapies [2,3]. These include both chemotherapy regimens and targeted therapies, optimized through a better understanding of the molecular carcinogenesis of colorectal cancer. Molecularly defined groups of colorectal cancers such as microsatellite instability (MSI)-high tumors and cancers with alterations in the receptor tyrosine kinase/KRAS/BRAF pathways, such as *KRAS*/*NRAS* or *BRAF* mutations and *ERBB2* amplifications, may benefit from targeted therapies [4,5,6]. Other metastatic colorectal cancers, such as microsatellite-stable cases and those with alterations in other pathways besides receptor tyrosine kinases/KRAS/BRAF, have fewer targeted options and are treated with cytotoxic chemotherapies or anti-angiogenic therapies [7,8].

Targeted therapies rely on the identification of and ability to reproducibly detect target alterations in clinical samples. This has led to the development of predictive molecular biomarkers, which are used to pair individual patient tumors with appropriate targeted treatments. Moreover, genomic classifications have been proposed, with the goals of defining prognostic groups of colorectal cancers and classifying cases according to genomic similarities, which may provide clues for therapeutic vulnerabilities. A consensus genomic classification of colorectal cancers based on a meta-analysis of six previously proposed classifications divides colorectal cancers into four consensus molecular sub-types, ranging from CMS1 to CMS4 [9]. The main characteristic of the CMS1 group is MSI. CMS2 represents the most abundant group and is characterized by CIN. It also shows increased WNT pathway signaling activity. The third genomic group, CMS3, features metabolic deregulation and frequent KRAS mutations. CMS4 is characterized by the activation of the Epithelial to Mesenchymal Transition (EMT), a process that facilitates tumor invasiveness, and by deregulation of the TGFβ pathway [9]. Some colorectal cancers display a mixture of characteristics from these four genomic groups and have been classified into a fifth mixed group. This consensus molecular classification underscores the key genomic properties within these groups and can help in identifying additional high-prevalence alterations with potential pathogenic importance that would otherwise be hidden in the bulk of unclassified colorectal cancers. The identification of therapeutic vulnerabilities nested in these alterations could also be facilitated within the more homogeneous sub-groups. Attempts to derive the information obtained by genomic approaches through alternative cheaper and clinically available methods, such as immunohistochemistry (IHC), have led to the proposal of a classification that partially captures the CMS genomic groups using IHC evaluations for the MMR-related proteins MSH2, MSH6, PMS2, and MLH1 and four other proteins, CDX2, FRMD6, HTR2B, and ZEB1 [10]. With this IHC-based schema, colorectal cancers with a loss of nuclear expression of one or more MMR-related proteins are classified into a group corresponding to the genomic CMS1 group. Among the rest, cancers with a retained expression of the transcription factor CDX2 (Caudal-related domain homeobox 2) are classified into an epithelial sub-type, corresponding to the genomic CMS2 and CMS3 groups, and colorectal cancers with a loss of CDX2 expression which also express FRMD6, HTR2B, and ZEB1 are classified into a mesenchymal sub-type, corresponding to the genomic CMS4 group. The IHC classification was confirmed to classify most (61 of 70) colorectal cancers into the correct corresponding genomic groups [10]. Expression of the CDX2 transcription factor, the main determinant of the epithelial sub-type, has been previously shown to be prognostic in stage II and III colorectal cancers [11]. About 10% of colorectal cancers were CDX2-negative and had a worse prognosis. In addition, patients with stage II disease and CDX2-negative expression derived benefits from adjuvant chemotherapies, while CDX2-positive stage II patients did not derive benefits [11].

In the current investigation, colorectal cancers with suppressed CDX2 mRNA expression are examined for concomitant molecular alterations and clinical characteristics and compared with colorectal cancers with no CDX2 suppression. The divergent landscape of these groups may become the basis for the successful development of targeted therapies.

## 2. Methods

Two publicly available genomic series, the colorectal cancer series from The Cancer Genome Atlas (TCGA) and the cohort from Sidra-LUMC AC-ICAM, which provide data on mRNA expressions, were selected for evaluation [12,13]. Groups of cancers with different levels of CDX2 mRNA expression were constructed and compared for their clinical and genomic characteristics, as well as molecular alterations of interest. The CDX2-suppressed group was defined with a cut-off of mRNA expression z-score relative to normal samples (log RNA Seq V2) of less than −2. The non-CDX2-suppressed group included samples with mRNA expression z-scores relative to normal samples (log RNA Seq V2) above 0.

Both TCGA and Sidra-LUMC AC-ICAM employed a whole-exome next-generation sequencing platform for their genomic analyses. The two studies provided data on mutations, copy number alterations, and structural variants. In TCGA, single-nucleotide mutation calling was conducted with inputs from various pipelines [14]. Copy number alterations were analyzed with the GISTIC (Genomic Identification of Significant Targets in Cancer) algorithm [15]. In the Sidra-LUMC AC-ICAM study, mutation calling was performed with the mutect (v.1.1.7) algorithm [13,16].

The TCGA and Sidra-LUMC AC-ICAM studies also assayed participating samples for mRNA expression. An algorithm called RSEM (RNA-Seq by Expectation Maximization), which does not require reference genome data, was used for the normalization of mRNA expression from RNA Seq data [17]. Chromosomal instability (CIN) was quantified with a score (Aneuploidy Score, AS) that was derived by summing the number of chromosome arms in each sample that had copy number alterations, either gains or losses. For the calculation of AS, chromosome arms were considered to be copy-number-altered if more than 80% of their length contained somatic copy number alterations. Chromosome arms with a lower burden of somatic copy number alterations extending from 20% to 80% of their length were considered to be indeterminate, and chromosome arms with somatic copy number alterations in less than 20% of their length were considered to be not altered. For the calculation of the AS from Affymetrix 6.0 SNP arrays, an algorithm called ABSOLUTE was used in TCGA [18]. The algorithm uses inputs of copy number data along with pre-calculated recurrent cancer karyotypes and point mutation data to provide an output of a quantitative ploidy estimation.

The interrogation of participating series at the individual case level from the two selected cohorts was performed online on the cBioPortal for Cancer Genomics platform (cBioportal, http://www.cbioportal.org). cBioportal is a site containing genomic and associated clinical data from publicly available studies, maintained by the Memorial Sloan Kettering Cancer Center (MSKCC) and other academic institutions [19,20].

The pathogenic implications of mutations in various cancer genes of interest in the CDX2-suppressed and non-CDX2-suppressed colorectal cancer groups were evaluated through the OncoKB knowledgebase [21]. OncoKB provides information on an extensive collection of cancer-related genes, which are classified as oncogenes or tumor suppressors. Each specific mutation in the genes curated in the database is designated as likely oncogenic, of unknown oncogenic effect, or likely neutral [21].

For comparisons of the categorical variables, statistical analyses were performed with the Fisher’s exact test or the χ^2^ test. The *t*-test test was used for the analysis of continuous variables. Overall survival (OS) evaluations were performed with the construction of Kaplan–Meier survival curves from source data. The log rank test was used to compare these Kaplan–Meier survival curves. All statistical comparisons were considered as significant if *p* < 0.05.

## 3. Results

### 3.1. The TCGA Colorectal Cancer Cohort

In the TCGA cohort, the CDX2-suppressed (CDX2 log RNA Seq V2 < −2) group of colorectal cancers consisted of 75 cases (12.6% of the entire TCGA colorectal cancer cohort) and the non-CDX2-suppressed (CDX2 log RNA Seq V2 > 0) group consisted of 249 cases (41.9% of the entire TCGA cohort). These two groups did not differ significantly in their mean age, percentage of patients above the age 65 years old, sex, or prevalence of advanced stages (Table 1). Non-CDX2-suppressed cancers were more commonly located in the rectum (30.1% of the cases), while CDX2-suppressed cancers were rectal in 12% of cases (Fisher’s exact test *p* = 0.001). The majority of CDX2-suppressed colorectal cancers (54.1%) were MSI high, while only a few cases of non-CDX2-suppressed cancers were MSI high (Table 2). Chromosomal instability (CIN) was the most prevalent characteristic in non-CDX2-suppressed cancers (88%) versus 37.8% of cases in the CDX2-suppressed group. Cases with a high TMB (above 10 mutations per Mb) were more frequent in the CDX2-suppressed group (53.8% versus 3.5% in the non-CDX2-suppressed group, Fisher’s exact test *p* < 0.0001). In contrast and consistent with the high frequency of CIN cancers in the non-CDX2-suppressed group, this group had a higher prevalence of cases with high AS and FGA scores (Table 2).

**Figure 1:**
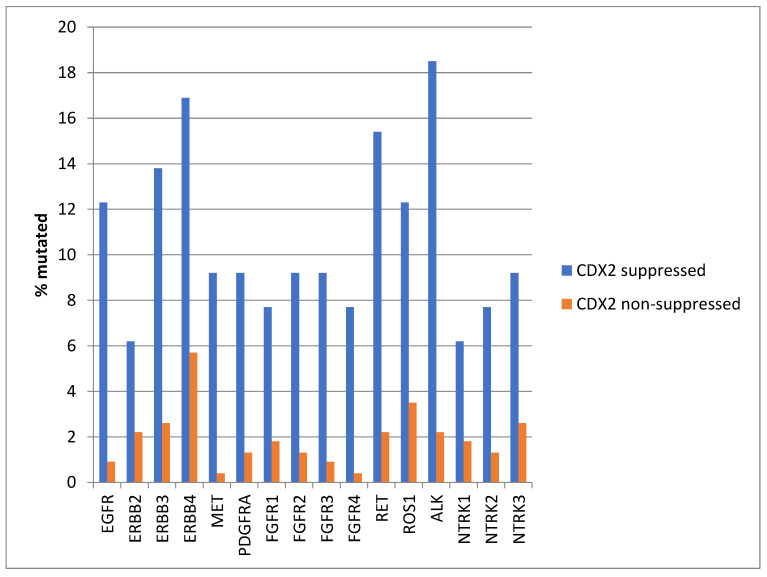
Prevalence of mutations in receptor tyrosine kinase genes in colorectal cancers with CDX2 suppression (mRNA expression z-scores relative to normal samples log RNA Seq V2 < −2) and non-CDX2-suppressed cancers (mRNA expression z-scores relative to normal samples log RNA Seq V2 > 0). Mutations in EGFR family members EGFR, ERBB3, and ERBB4, as well as mutations in RET, ROS1, and ALK showed a prevalence above 12% in CDX2-suppressed colorectal cancers. Data are from TCGA.

Mutations in several receptor tyrosine kinases, including the EGFR family members EGFR, ERBB3, and ERBB4, the kinases MET and PDGFRA, the FGFR family members FGFR1, FGFR2, FGFR3, and FGFR4, the kinases RET, ROS1, and ALK, and the NTRK family members NTRK2 and NTRK3, were significantly more prevalent in CDX2-suppressed colorectal cancers (Figure 1). All these kinases displayed mutations in more than 6% of CDX2-suppressed colorectal cancers in the TCGA cohort, and some receptor tyrosine kinases, including EGFR, ERBB3, ERBB4, RET, ROS1, and ALK, showed even higher mutation rates in these cancers (Figure 1).

**Figure 2:**
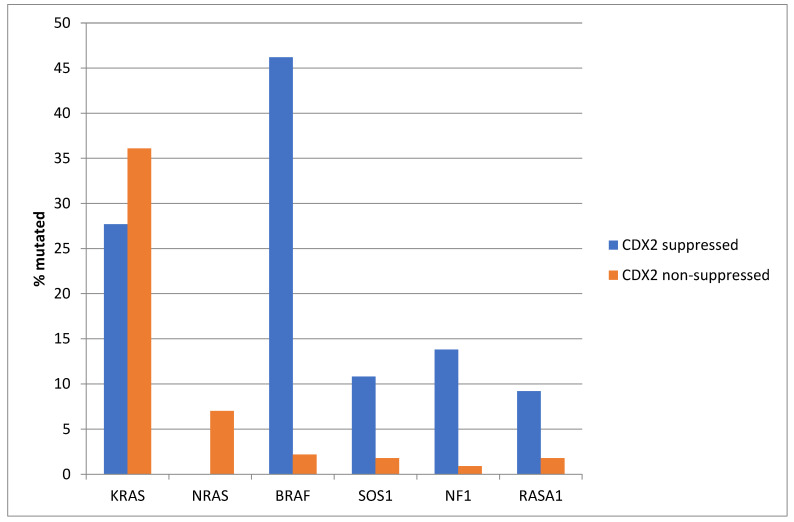
Prevalence of KRAS/BRAF pathway mutations in colorectal cancers with CDX2 suppression (mRNA expression z-scores relative to normal samples log RNA Seq V2 < −2) and non-CDX2-suppressed cancers (mRNA expression z-scores relative to normal samples log RNA Seq V2 > 0). Mutations in the two RAS homologues are more prevalent in non-CDX2-suppressed cancers and BRAF mutations, as well as SOS1, NF1, and RASA1 mutations are significantly more prevalent in CDX2-suppressed colorectal cancers. Data are from TCGA.

The prevalence of *KRAS* mutations was not significantly different between the CDX2-suppressed group, which showed a *KRAS* mutation rate of 27.7%, and the non-CDX2-suppressed group, which showed a *KRAS* mutation rate of 36.1% (Fisher’s exact test *p =* 0.23, Figure 2). Mutations in the related *NRAS* gene were observed in 7% of non-CDX2-suppressed colorectal cancers and in no cases of the CDX2-suppressed group (Fisher’s exact test *p =* 0.02). In contrast to the two RAS genes, mutations in other genes of the RAS/RAF/MEK pathway were more prevalent in CDX2-suppressed colorectal cancers (Figure 2). *BRAF* mutations were common in CDX2-suppressed colorectal cancers (46.2%), but rare in non-CDX2-suppressed cancers (2.2%, Fisher’s exact test *p* < 0.0001). *SOS1*, *RASA1*, and *NF1* mutations were also significantly more often observed in CDX2-suppressed cancers (10.8%, 9.2% and 13.8%, respectively) than in non CDX2-suppressed cancers (1.8%, 1.8%, and 0.9%, Fisher’s exact test *p =* 0.003, 0.009, and 0.0001, respectively, Figure 2).

**Figure 3:**
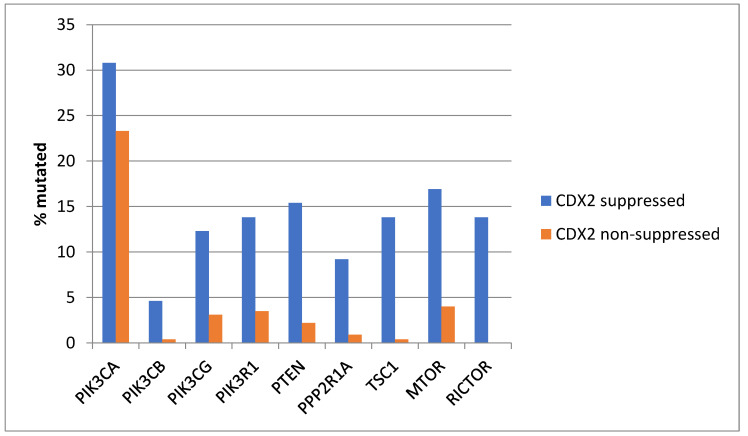
Prevalence of PI3K/AKT/mTOR pathway mutations in colorectal cancers with CDX2 suppression (mRNA expression z-scores relative to normal samples log RNA Seq V2 < −2) and non-CDX2-suppressed cancers (mRNA expression z-scores relative to normal samples log RNA Seq V2 > 0). Mutations in PIK3CA were not significantly different between the two groups, but several other pathway genes were significantly more frequently mutated in CDX2-suppressed cancers. Data are from TCGA.

Genes encoding for several components of the PI3K/AKT pathway were more commonly mutated in CDX2-suppressed colorectal cancers (Figure 3). In colorectal cancer, the alpha catalytic sub-unit of the PI3K kinase gene is frequently mutated, and *PIK3CA* was mutated in 30.8% of CDX2-suppressed colorectal cancers compared to 23.3% of non-CDX2-suppressed colorectal cancers (Fisher’s exact test *p =* 0.25). The genes encoding for the beta (*PIK3CB*) and gamma (*PIK3CG*) catalytic sub-units and the regulatory sub-unit 1 (*PIK3R1*) of PI3K were more commonly mutated in CDX2-suppressed colorectal cancers (Figure 3). Mutations in other components of the pathway, including PTEN, TSC1, PPP2R1A, and MTOR, were also significantly more frequent in CDX2-suppressed colorectal cancers (Figure 3).

**Figure 4:**
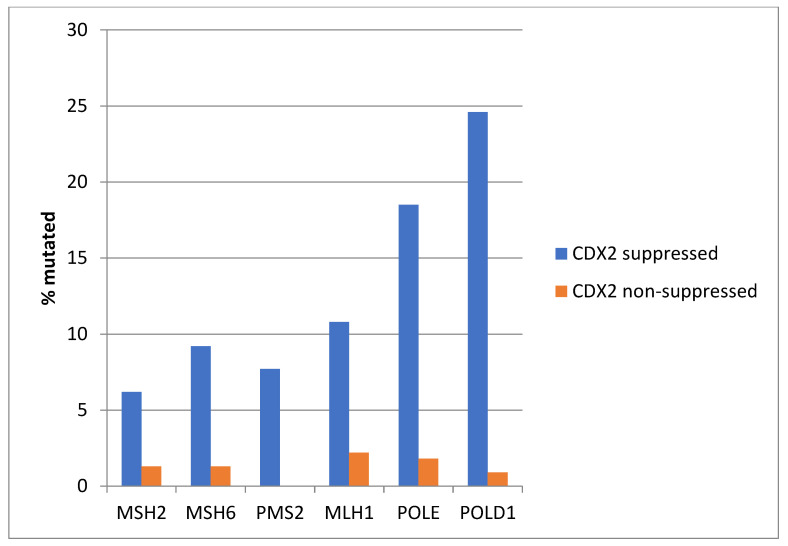
Mutations in mismatch repair associated proteins MSH2, MSH6, MLH1, and PMS2 and the proofreading polymerases POLE and POLD1 were more prevalent in colorectal cancers with CDX2 suppression (mRNA expression z-scores relative to normal samples log RNA Seq V2 < −2) compared with non-CDX2-suppressed cancers (mRNA expression z-scores relative to normal samples log RNA Seq V2 > 0). Data are from TCGA.

Consistent with the high prevalence of MSI-high cancers in the CDX2-suppressed group, genes encoding for the MMR-associated proteins MSH2, MSH6, PMS2, and MLH1 were significantly more frequently mutated in this group compared with the non-CDX2 -suppressed group (Figure 4). Similarly, the genes encoding for the proofreading polymerases POLE and POLD1 displayed significantly higher mutation rates in CDX2-suppressed colorectal cancers (Figure 4).

**Figure 5:**
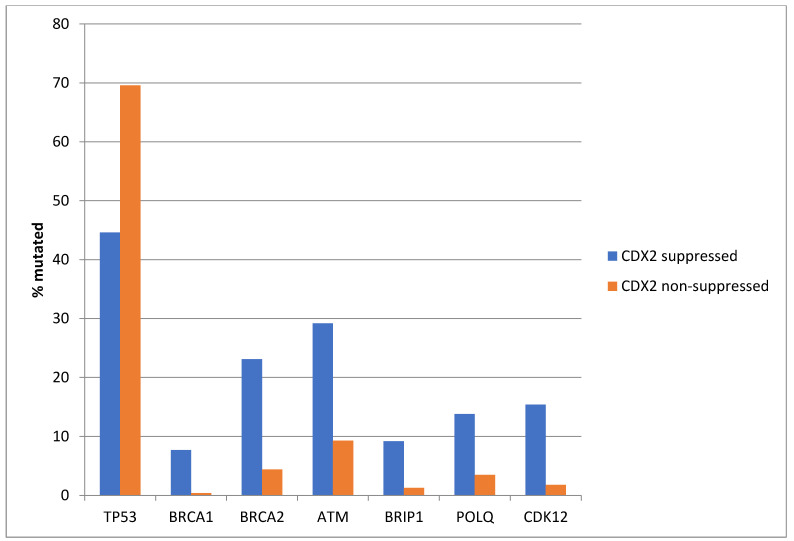
Mutations in TP53 were significantly more prevalent in non-CDX2-suppressed cancers (mRNA expression z-scores relative to normal samples log RNA Seq V2 > 0) compared with CDX2-suppressed (mRNA expression z-scores relative to normal samples log RNA Seq V2 < −2) cancers. In contrast, other DNA-damage-response-associated genes were more frequently mutated in CDX2-suppressed colorectal cancers, with the rate of mutations in BRCA2 and ATM exceeding 20% in this group. Data are from TCGA.

Among the genes involved in DNA damage response and repair, *TP53* is often mutated in colorectal cancers and was more often mutated in non-CDX2-suppressed cases (69.6%) compared with CDX2-suppressed cases (44.6%, Fisher’s exact test *p =* 0.0004, Figure 5). In contrast, other DNA damage response and repair genes, including *BRCA1*, *BRCA2*, *ATM*, *BRIP*, *POLQ*, and *CDK12*, were significantly more often mutated in CDX2-suppressed cancers (Figure 5).

**Figure 6:**
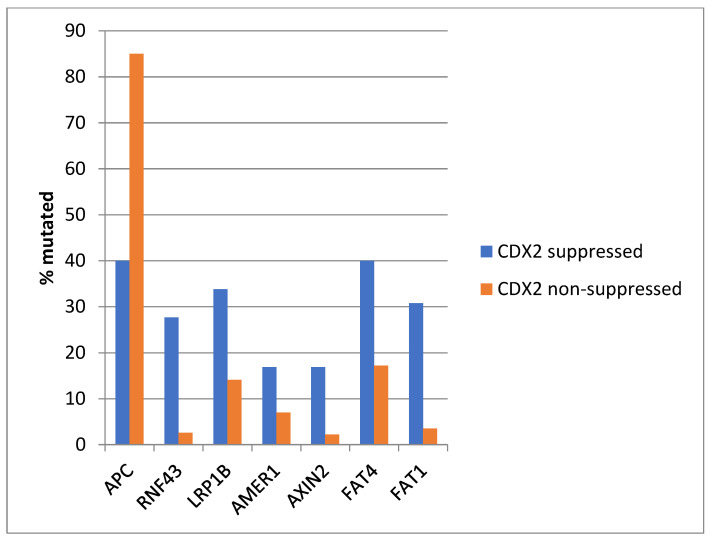
Mutations in APC were significantly more prevalent in non- CDX2-suppressed cancers (mRNA expression z-scores relative to normal samples log RNA Seq V2 > 0) compared with CDX2-suppressed (mRNA expression z-scores relative to normal samples log RNA Seq V2 < −2) cancers. In contrast, other genes of the WNT/APC/β-catenin pathway, including RNF43, LRP1B, AMER1, AXIN2, and the atypical cadherins FAT1 and FAT4, were significantly more frequently mutated in CDX2-suppressed colorectal cancers. Data are from TCGA.

The most frequently mutated gene of the WNT/β-catenin pathway in colorectal cancers, *APC* was more often mutated in the non- CDX2-suppressed group (85% of the cases) compared with the CDX2-suppressed group (APC mutations in 40% of cases, Fisher’s exact test *p* < 0.0001, Figure 6). Genes encoding for several other components of the WNT/β-catenin pathway (*RNF43*, *LRP1B*, *AMER1*, *AXIN2*, *FAT4*, and *FAT1*) were significantly more often mutated in CDX2-suppressed cases (Figure 6).

**Figure 7:**
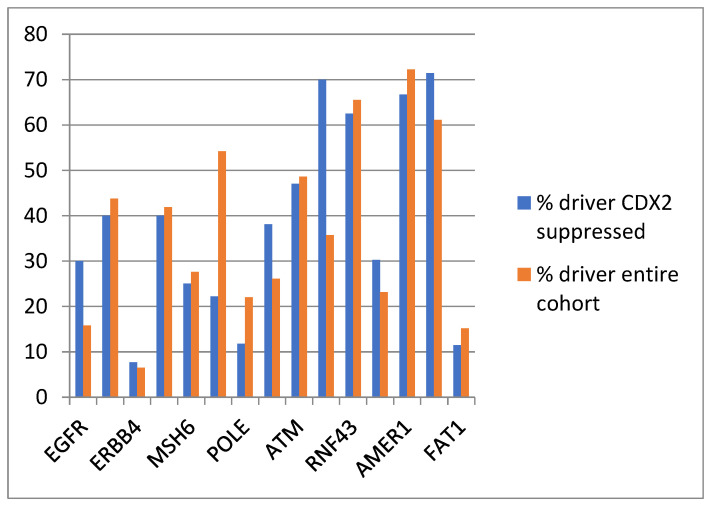
Prevalence of driver mutations in the entire TCGA colorectal cancer cohort and in the group with CDX2-suppressed (mRNA expression z-scores relative to normal samples log RNA Seq V2 < −2) colorectal cancers from the TCGA cohort.

The evaluation of the functional repercussions of mutations in cancer-pathway-associated genes showed that mutations occurring in the CDX2-suppressed group were potentially pathogenic in similar percentages, or for some genes, in higher percentages than mutations of the same genes in the entire TCGA colorectal cancer cohort (Figure 7 and Table 3). The most frequent copy number alteration in colorectal cancer, amplification at locus 20q11, was observed in 12.5% to 12.9% of non-CDX2-suppressed cancers, but in none of the CDX2-suppressed cases (Fisher’s exact test *p =* 0.0004).

**Figure 8:**
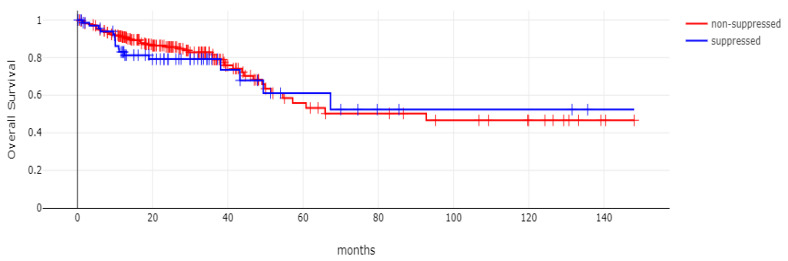
Overall survival (OS) of patients with CDX2-suppressed colorectal cancers versus non-CDX2-suppressed in the TCGA cohort (Log Rank test *p =* 0.53).

**Figure 9:**
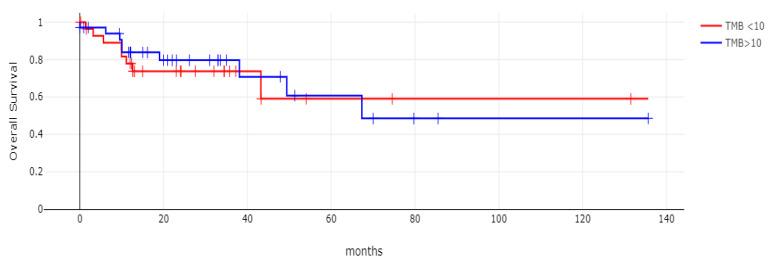
Overall survival (OS) of the sub-groups with high TMB (above 10 mutations/Mb) or low TMB within the CDX2-suppressed group (Log Rank test *p =* 0.72). Data are from TCGA.

**Figure 10:**
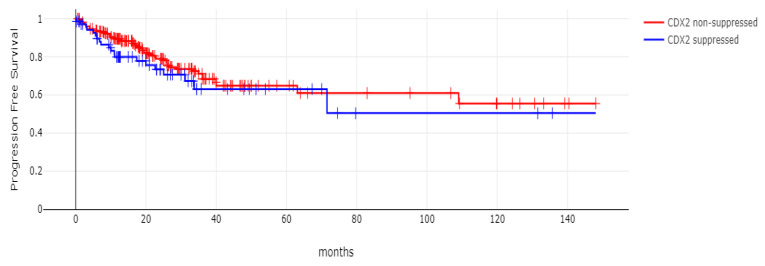
Progression-free survival (PFS) of patients with CDX2-suppressed colorectal cancers versus non-CDX2-suppressed in the TCGA cohort (Log Rank test *p =* 0.29).

**Figure 11:**
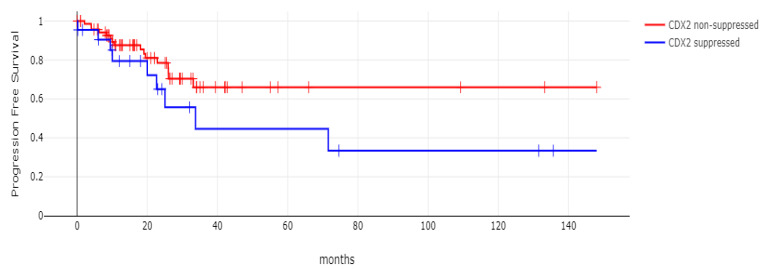
Progression-free survival (PFS) of stage III colorectal cancer patients with CDX2-suppressed colorectal cancers versus non-CDX2-suppressed. A non-significant trend for worse PFS for stage III patients with CDX2-suppressed cancer was observed (Log Rank test *p =* 0.12). Data are from TCGA.

With 66 events recorded in the 308 patients participating in the survival analysis, the OS of patients with CDX2 suppression did not differ significantly from the OS of those without CDX2 suppression in the TCGA cohort (Log Rank test *p =* 0.53, Figure 8). The median follow-up of patients alive was 30.1 months. The OS of the sub-groups with a high TMB (above 10 mutations/Mb) or low TMB within the CDX2-suppressed group also did not differ (Log Rank test *p =* 0.72, Figure 9). The progression-free survival of the two groups, with and without CDX2 suppression, was also not significantly different (Log Rank test *p =* 0.29, Figure 10). A non-significant trend for a worse PFS for stage III patients with CDX2-suppressed cancer was observed (Log Rank test *p =* 0.12, Figure 11).

### 3.2. The Sidra-LUMC AC-ICAM Cohort

The Sidra-LUMC AC-ICAM cohort contained a lower percentage of colon cancers with CDX2 suppression (6%, 21 of 348 cases) and a higher percentage of non-CDX2-suppressed cases (62.9%, 219 of 348 cases) than TCGA. Similar to TCGA, CDX2-suppressed and non-CDX2-suppressed colon cancers did not differ significantly in their mean age, percentage of patients above 65 years old, or prevalence of metastatic disease (Table 4). Primary location in the right colon was observed in 81% of CDX2-suppressed cancers, while only 43.4% of non-CDX2-suppressed cancers were located in the right colon (Fisher’s exact test *p =* 0.001). CDX2-suppressed cancers were predominantly (66.7% of cases) of the CMS1 consensus molecular sub-type and less frequently belonged to CMS4, while no cases of CMS2 and CMS3 were included in the group. In contrast, non-CDX2-suppressed colon cancers were most commonly of the CMS2 (33.8%) and CMS3 (23.7%) sub-types, less frequently (18.3%) of the CMS4 sub-type, and rarely belonged to CMS1 (Table 4). Similar to TCGA, CDX2-suppressed cancers showed more commonly (68.8%) a low CIN, as measured by the FGA score and a high TMB above 10 mutations/Mb, than non-CDX2-suppressed cancers.

**Figure 12:**
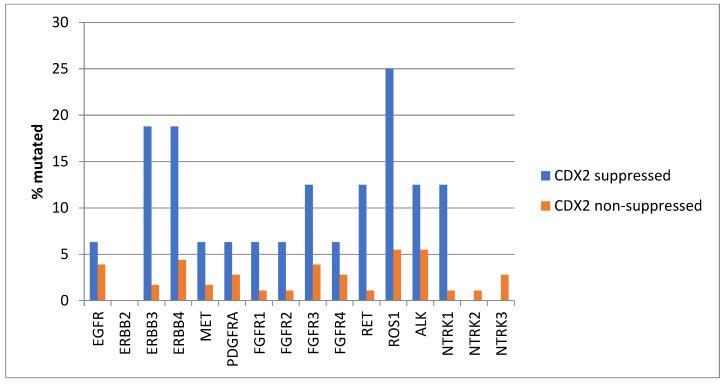
Prevalence of mutations in receptor tyrosine kinase genes in colorectal cancers with CDX2 suppression (mRNA expression z-scores relative to normal samples log RNA Seq V2 < −2) and non-CDX2-suppressed cancers (mRNA expression z-scores relative to normal samples log RNA Seq V2 > 0). Mutations in EGFR family members ERBB3 and ERBB4, as well as mutations in RET, ROS1, and NTRK1, showed statistically significant higher prevalence in CDX2-suppressed colorectal cancers. Data are from the Sidra-LUMC AC-ICAM cohort.

Genes encoding for several receptor tyrosine kinases were mutated in a significant minority of CDX2-suppressed colon cancers, while the prevalence of such mutations was lower in most of these genes in the group with non-CDX2-suppressed colon cancers (Figure 12). Mutations in *ERBB3*, *ERBB4*, *RET*, *ROS1*, and *NTRK1* were statistically significantly more prevalent in the CDX2-suppressed group.

**Figure 13:**
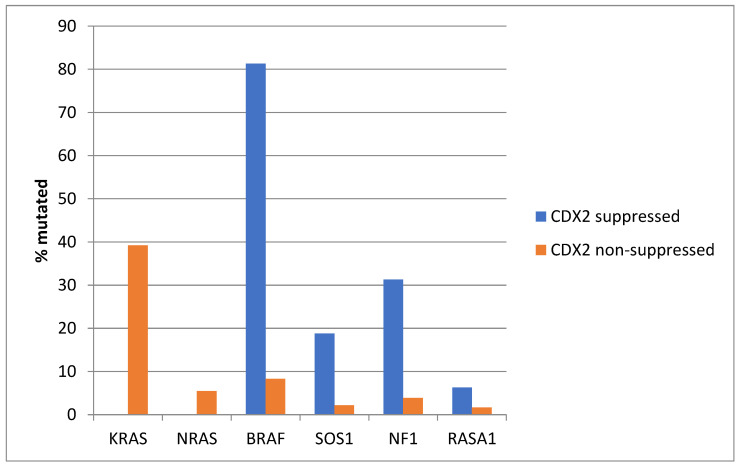
Prevalence of KRAS/BRAF pathway mutations in colorectal cancers with CDX2 suppression (mRNA expression z-scores relative to normal samples log RNA Seq V2 < −2) and non-CDX2-suppressed cancers (mRNA expression z-scores relative to normal samples log RNA Seq V2 > 0). Mutations in the two RAS homologues are more prevalent in non-CDX2-suppressed cancers and BRAF mutations, as well as SOS1 and NF1 mutations, are significantly more prevalent in CDX2-suppressed colorectal cancers. Data are from the Sidra-LUMC AC-ICAM cohort.

**Figure 14:**
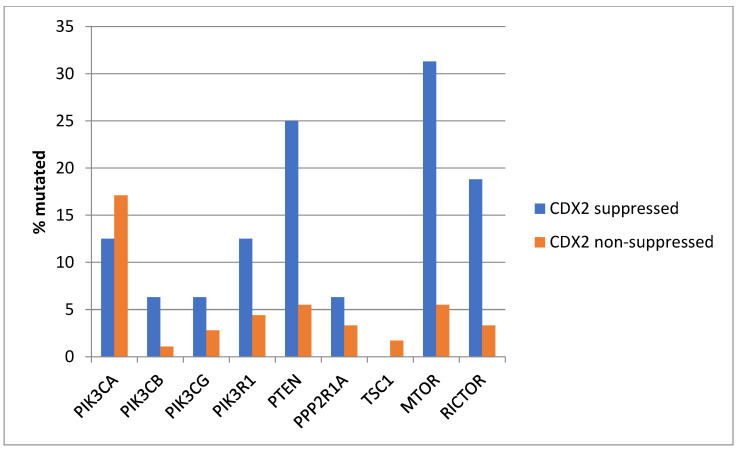
Prevalence of PI3K/AKT/mTOR pathway mutations in colorectal cancers with CDX2 suppression (mRNA expression z-scores relative to normal samples log RNA Seq V2 < −2) and non-CDX2-suppressed cancers (mRNA expression z-scores relative to normal samples log RNA Seq V2 > 0). Mutations in PIK3CA were not significantly different between the two groups, but several other pathway genes, including PTEN, MTOR, and RICTOR, were significantly more frequently mutated in CDX2-suppressed cancers. Data are from the Sidra-LUMC AC-ICAM cohort.

*KRAS* and *NRAS* mutations were observed exclusively in non-CDX2-suppressed colon cancers, with no cases in the CDX2-suppressed group bearing such mutations (Figure 13). In contrast, mutations in three other genes of the KRAS pathway, *BRAF*, *SOS1*, and *NF1*, were significantly more common in CDX2-suppressed cancers. In addition, several genes of the parallel PI3K/AKT pathway were more frequently mutated in CDX2-suppressed cancers, with the differences for *PTEN*, *MTOR,* and *RICTOR* reaching significance (Figure 14).

**Figure 15:**
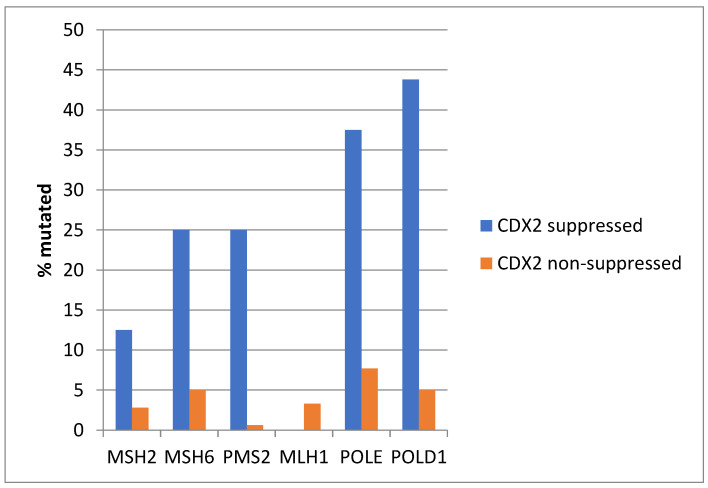
Mutations in mismatch-repair-associated proteins MSH6 and PMS2 and the proofreading polymerases POLE and POLD1 were more prevalent in colorectal cancers with CDX2 suppression (mRNA expression z-scores relative to normal samples log RNA Seq V2 < −2) compared with non-CDX2-suppressed cancers (mRNA expression z-scores relative to normal samples log RNA Seq V2 > 0). Data are from the Sidra-LUMC AC-ICAM cohort.

Among MMR-associated genes, *MSH6* and *PMS2* were significantly more frequently mutated in CDX2-suppressed colon cancers, and the same was true for mutations in the proofreading polymerases *POLE* and *POLD1* genes (Figure 15).

**Figure 16:**
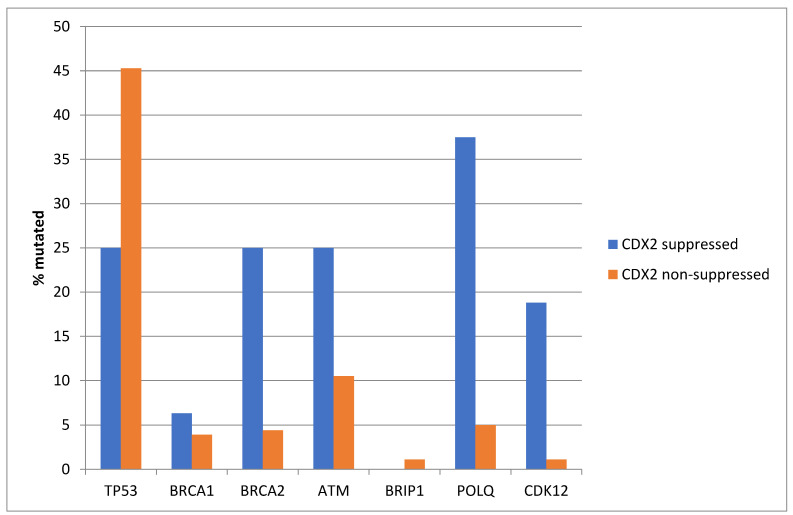
Mutations in TP53 were significantly more prevalent in non-CDX2-suppressed cancers (mRNA expression z-scores relative to normal samples log RNA Seq V2 > 0) compared with CDX2-suppressed (mRNA expression z-scores relative to normal samples log RNA Seq V2 < −2) cancers. In contrast, other DNA-damage-response-associated genes were more frequently mutated in CDX2-suppressed colorectal cancers, with the rate of mutations in BRCA2, POLQ, and CDK12 reaching statistical significance. Data are from the Sidra-LUMC AC-ICAM cohort.

Similar to TCGA, *TP53* mutations were more prevalent in the non-CDX2-suppressed group of the Sidra cohort (45.3%) compared with 25% in the CDX2-suppressed group (Fisher’s exact test *p =* 0.02, Figure 16). In contrast, and also consistent with the TCGA results, several other DDR-related genes were more frequently mutated in the CDX2-suppressed group, with differences for *BRCA2*, *CDK12*, and *POLQ* reaching statistical significance (Figure 16).

**Figure 17:**
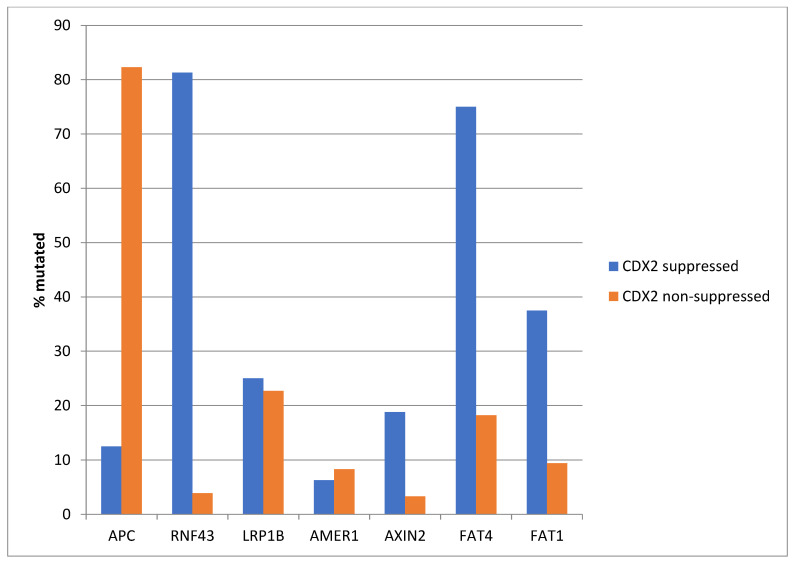
Mutations in APC were significantly more prevalent in non-CDX2-suppressed cancers (mRNA expression z-scores relative to normal samples log RNA Seq V2 > 0) compared with CDX2-suppressed (mRNA expression z-scores relative to normal samples log RNA Seq V2 < −2) cancers. In contrast, other genes of the WNT/APC/β-catenin pathway, including RNF43, AXIN2, and the atypical cadherins FAT1 and FAT4, were significantly more frequently mutated in CDX2-suppressed colorectal cancers. Data are from the Sidra-LUMC AC-ICAM cohort.

Similar to the TCGA pattern, *APC* mutations were significantly more common in the non CDX2-suppressed group of the Sidra-LUMC AC-ICAM cohort (82.3% versus 12.5%, Fisher’s exact test *p* < 0.0001, Figure 17). Mutations in other genes of the WNT/β-catenin pathway were more prevalent in the CDX2-suppressed group, with the most significant differences observed in *RNF43*, which was mutated in 81.3% of the cases in this group versus 3.9% of the cases in non-CDX2-suppressed cancers (Fisher’s exact test *p* < 0.0001, Figure 1), and in atypical cadherin FAT4, which was mutated in 75% of the cases in the CDX2-suppressed group versus 18.2% of the cases in non-CDX2-suppressed cancers (Fisher’s exact test *p* < 0.0001, Figure 17).

Similar to TCGA, the OS and PFS of patients with CDX2-suppressed colon cancers in the Sidra-LUMC AC-ICAM cohort were not different from the OS and PFS of patients with non-CDX2-suppressed colon cancers (Log Rank test *p =* 0.53 and 0.4, respectively), although the numbers in the cohorts and events were lower.

## 4. Discussion

Caudal-related homeobox transcription factor 2 (CDX2) is a transcription factor with a role in the development and specification of the midgut and hindgut [22,23]. During embryonic development, CDX2 is initially expressed in trophectoderm in the early zygote and then becomes localized in midgut and hindgut endoderm, while the expression of transcription factor SOX2 predominates more proximally, specifying the foregut endoderm that will give rise to the stomach [24]. CDX2 is expressed in the lower adult human gastrointestinal tract with the highest level of expression in the right colon and a lower expression in both the distal colon and the small intestine [25]. In the colonic epithelium, CDX2 is expressed in both proliferating crypt cells and in differentiated intestinal epithelial cells across the villi [22]. The expression of CDX2 in IHC sections is used in clinical pathology for the confirmation of the colonic origin of cancers in biopsies, usually in combination with cytokeratins CK20, which is usually co-expressed in the colon, and CK7, which is not expressed [26]. About 90% of colon cancers express CDX2 and have a better prognosis than CDX2-negative colon cancers [11].

The expression and function of CDX2 are regulated at the transcriptional and post-transcriptional levels. The transcription factor function of CDX2 leads to the trans-activation of key genes for intestinal physiology [25]. Among well-established CDX2 targets, the enzymes sucrose isomaltase and carbonic anhydrase 1 are critical for the absorptive intestinal function. The iron transport protein hephaestin is also a target of CDX2 [27]. Mucin 2 is another target of CDX2 and contributes to the protective barrier of the intestinal epithelium. Adhesion proteins claudin 2 and cadherin 17 are also among CDX2 targets as part of its maintenance of epithelial integrity function.

In the current investigation, the group of colorectal cancers with CDX2 suppression was confirmed to contain a high percentage of MSI-high tumors and tumors with a TMB above 10 mutations/Mb. In addition, about two-thirds of these cancers belong to the CMS1 genomic category, which was only rarely observed in the non-CDX2-suppressed group. The rest of the CDX2-suppressed cancers belonged to the CMS4 group, which was also observed in a sizeable minority (18.3%) of non-CDX2-suppressed tumors. The CDX2-suppressed group possessed mutations in several receptor tyrosine kinases and down-stream KRAS/BRAF/MEK and PI3K/AKT pathways, except for KRAS mutations, which were more prevalent in the non-CDX2-suppressed group. Consistent with the results of the current study, another recent study confirmed the association of a CDX2 loss of protein expression by immunohistochemistry, as well as the loss of another transcription factor expressed in the colon, SATB2, with mismatch repair deficiency and BRAF mutations [28]. Another report pinpointed to the association of CDX2 suppression with right colon cancers and BRAF mutations [29]. This study showed that the loss of *cdx2* in mice affected the epithelial phenotype of cells of proximal colon organoids and promoted Wnt pathway independent growth, synergizing with mutant *braf*. Therefore, CDX2 programs may safeguard the epithelial identity of the colonic epithelium, and losses of these programs promote the mesenchymal transition, as observed in a subset of colon samples belonging to the CMS4 genomic group.

Besides TP53 and APC mutations, which were both more common in non-CDX2-suppressed colorectal cancers, CDX2-suppressed cancers had a higher prevalence of mutations in other DDR-related and WNT/APC/β-catenin pathway genes. These landscapes suggest that CDX2-suppressed and non-CDX2-suppressed colorectal cancers use different molecular alterations to activate the same cancer-associated pathways. The implications for pertinent tailored targeted therapeutic approaches in these two groups can be envisioned. Targeting mutated receptor tyrosine kinases in CDX2-suppressed cancers may be a feasible therapeutic strategy, although not all alterations discovered are currently matched with effective inhibitors. As allosteric mutation specific inhibitors have been discovered, a detailed understanding of receptor mutations at the individual level will be required for advancing the field, similar to the *KRAS* mutations, where inhibitors of G12C mutations have been introduced [5]. Other therapeutic opportunities in CDX2-suppressed colorectal cancers arise for the sub-set that are MSI high or have a high TMB, which could be candidates for immunotherapy with immune checkpoint inhibitors, and for the *BRAF* mutated sub-set, could be treated with BRAF inhibitor combinations with anti-EGFR monoclonal antibodies [4,6]. Common mutations in DDR-associated genes, such as *BRCA2* and *ATM*, in CDX2-suppressed cancers may offer additional therapeutic opportunities for treatment with PARP inhibitors or inhibitors of ATM that are in development [30,31]. Inhibitors of the WNT/APC/β-catenin pathway are not currently clinically available, but several are in development, including porcupine inhibitors and tankyrase inhibitors [32,33]. The sensitivity of cancers with alternatives to APC mutations in the WNT/APC/β-catenin pathway remains to be determined as these inhibitors progress to clinical trials.

Despite a low prevalence of APC mutations compared with non-CDX2-suppressed colorectal cancers, CDX2-suppressed cancers showed a higher prevalence of mutations in several other components of the WNT/APC/β-catenin pathway. The tumor suppressor ubiquitin ligase RNF43 (RING finger protein 43), one of the protein components of the pathway with a high mutation prevalence in CDX2-suppressed colorectal cancers, is a down-regulator of the Frizzled receptors of the pathway, through ubiquitination leading to the proteasome degradation of the receptors and decreased pathway activity [34]. The mutual exclusivity of RNF43 and APC mutations observed in the current report was previously reported in colorectal cancer in a report that did not examine CDX2 suppression [35]. Although loss of function mutations in RNF43 are currently not directly targetable, they have been associated with the response to anti-EGFR/BRAF combination therapies in microsatellite-stable BRAF-mutated colorectal cancers [36]. Microsatellite-stable BRAF-mutated metastatic colorectal cancer patients treated with anti-EGFR/BRAF combinations had better responses and survival outcomes when RNF43 was mutated compared with wild-type cases. RNF43 mutation status was not predictive of response to treatment with chemotherapy [35]. Mutations in other components of the WNT/APC/β-catenin pathway, including *APC* and *CTNNB1*, were also not associated with anti-EGFR/BRAF therapy benefits, suggesting that RNF43 function interferes with anti-EGFR/BRAF drugs’ therapeutic efficacy through WNT, β-catenin-independent, alternative pathway signaling or through other ubiquitination targets. The planar cell polarity pathway is important for epithelial cell positioning in cell membranes and is a pathway where WNT signals independently of β-catenin and its destruction complex [37]. Therefore, it could be affected by mutations in RNF43, leading to a loss of epithelial polarity and invasiveness. Interestingly, alternative ubiquitination targets of RNF43 and homologous ligase ZNRF3 at the plasma membrane include EGFR, which is deregulated by pathogenic mutations in a sub-set of CDX2-suppressed colorectal cancers [38]. A loss of RNF43 ubiquitination function due to mutations would allow for the stabilization and increased signaling of the receptor kinase. Whether other member of the EGFR family or other receptor tyrosine kinases that are also frequently mutated in CDX2-suppressed colorectal cancers are also targets of RNF43 is currently unknown. The EGFR family and other receptor tyrosine kinases are targets of ubiquitination by other ubiquitin ligases, and ubiquitination constitutes a process leading to their endocytosis [39].

In contrast to previous results that have shown worse prognoses for stage II and III CDX2-negative colorectal cancers compared with CDX2-positive cancers, the current analysis did not disclose significant differences in survival outcomes [11,40]. Other investigations have suggested that the prognostic value of the lack of CDX2 expression is restricted in the CMS4 group and not seen in CMS1 cancers [41]. However, no significant differences were observed in the OS of the subgroups of CDX2-suppressed patients with a high and low TMB (as a surrogate of MSI and CMS1) in TCGA. These divergent results may be due to the limited overall number of patients in the CDX2-suppressed group and small number of survival events, as well as the inclusion of patients across stages. Consistent with this hypothesis, a non-significant trend for a worse PFS in the CDX2-suppressed group was observed when the analysis was restricted to stage III patients. Moreover, reports that suggested that a loss of CDX2 is an adverse prognostic factor in colorectal cancer employed, in contrast to the current work that was based on mRNA expression, protein analysis by immunohistochemistry [11,40].

## 5. Conclusions

In conclusion, the group of colorectal cancers with CDX2 mRNA suppression represents a sub-set of the disease with a landscape offering opportunities for therapeutic targeting. Although this group of mRNA-suppressed cases may not completely overlap with colorectal cancers that show CDX2 loss at the protein level by IHC, a close approximation should allow for the use of IHC for defining the group in clinical practice and, subsequently, assaying for alterations with clinical targetability as specific inhibitors are introduced in the clinic. Pre-clinical work in cell lines and xenografts with the relevant molecular alterations may guide the development of these drugs in appropriate subsets of colorectal cancers possessing these alterations. In these experiments, the gain of function and loss of function manipulation of specific genes with potential relevance for the examined drug activity could be used to shed light on the mechanism of action and promote rational and personalized developments.

## Figures and Tables

**Table 1:** Characteristics of colorectal cancers with suppressed CDX2 [z-score relative to normal samples (log RNA Seq V2) below −2] and non-suppressed CDX2 [z-score relative to normal samples (log RNA Seq V2) above 0]. Data are from TCGA. Percentages are shown in parentheses. NA: Not available.

Characteristic	Entire Cohort (n = 594)	CDX2 Suppressed (n = 75)	Non CDX2 Suppressed (n = 249)	*p*
Age (mean +/− SD)	66.1 +/− 13.4	64.4 +/− 15.3	66.6 +/− 12.5	0.2
Age				
≤65 years-old	260 (43.9)	36 (48)	106 (42.7)	0. 42
>65 years-old	332 (56.1)	39 (52)	142 (57.3)	
NA	2		1	
Sex				
Male	312 (52.7)	36 (48)	139 (56)	0. 23
Female	280 (47.3)	39 (52)	109 (44)	
NA	2		1	
stage				
I	104 (17.9)	7 (9.6)	40 (16.4)	I–II versus III–IV
II	220 (37.9)	36 (49.3)	90 (36.9)	0.35
III	170 (29.3)	22 (30.1)	74 (30.3)	
IV	86 (14.8)	8 (11)	40 (16.4)	
NA	14	2	5	
Location primary				
Colon	436 (74.1)	66 (88)	174 (69.9)	0.001
Rectal	152 (25.9)	9 (12)	75 (30.1)	
NA	6			

**Table 2:** Subtype, Tumor Mutation Burden (TMB), Aneuploidy Score (AS), and Fraction Genome Altered (FGA) in colorectal cancers with suppressed (z-score relative to normal samples (log RNA Seq V2) below −2) and non-suppressed CDX2 (z-score relative to normal samples (log RNA Seq V2) above 0) from TCGA. Percentages are shown in parentheses. NA: Not available.

Characteristic	Entire Cohort (n = 594)	CDX2 Suppressed (n = 75)	Non CDX2 Suppressed (n = 249)	*p*
Subtype				
GS	58 (12.7)	3 (4.9)	20 (10)	GS versus CIN versus MSI
CIN	328 (71.4)	23 (37.8)	176 (88)	<0.0001
MSI	63 (13.7)	33 (54.1)	3 (1.5)	
POLE	10 (2.2)	2 (3.2)	1 (0.5)	
NA	135	14	49	
TMB				
≤10	451	30 (46.2)	219 (96.5)	<0.0001
>10	83	35 (53.8)	8 (3.5)	
NA	60	10	22	
AS				
<4	108 (18.4)	34 (45.9)	16 (6.5)	<0.0001
4–24	427 (72.9)	36 (48.7)	211 (85.1)	
>24	51 (8.7)	4 (5.4)	21 (8.4)	
NA	8	1	1	
FGA				
<0.08	118 (20.2)	19 (25.3)	15 (6.1)	<0.0001
0.08–0.35	316 (54.2)	44 (58.7)	77 (31.6)	
>0.35	149 (25.6)	12 (16)	152 (62.3)	
NA	11		5	

**Table 3:** Overall mutation frequencies and frequencies of putative driver mutations in representative genes with higher prevalence in CDX2-suppressed samples in the entire TCGA cohort and in the group with CDX2 suppression. Data for driver mutation status are from the OncoKB knowledgebase.

Gene	Entire Cohort (n = 594) (%)	Putative Drivers (%)	CDX2 Suppressed (n = 75) (%)	Putative Drivers (%)	*p*
EGFR	19 (3.2)	3 (0.5)	10 (13.3)	3 (4)	0.34
ERBB3	32 (5.4)	14 (2.4)	10 (13.3)	4 (5.3)	1
ERBB4	62 (10.4)	4 (0.7)	13 (17.3)	1 (1.3)	1
FGFR2	22 (3.7)	2 (0.3)	11 (14.7)	1 (1.3)	1
FGFR3	18 (3)	1 (0.2)	6 (8)	0	1
RET	30 (5.1)	2 (0.3)	12 (16)	2 (2.7)	0.56
ALK	41 (6.9)	1 (0.2)	14 (18.7)	0	1
NTRK3	29 (4.9)	3 (0.5)	9 (12)	1 (1.3)	1
BRAF	67 (11.3)	56 (9.4)	33 (44)	30 (40)	0.37
NF1	43 (7.2)	18 (3)	15 (20)	6 (8)	1
RASA1	29 (4.9)	8 (1.3)	10 (13.3)	1 (1.3)	0.39
PIK3CB	16 (2.7)	2 (0.3)	3 (4)	0	1
PIK3R1	38 (6.4)	29 (4.9)	11 (14.7)	9 (12)	1
PTEN	48 (8.1)	46 (7.7)	15 (20)	15 (20)	1
PPP2R1A	15 (2.5)	6 (1)	6 (8)	2 (2.7)	1
TSC1	21 (3.5)	7 (1.2)	11 (14.7)	6 (8)	0.28
MTOR	46 (7.7)	5 (0.8)	14 (18.7)	0	0.32
MSH2	27 (4.5)	10 (1.7)	7 (9.3)	4 (5.3)	0.41
MSH6	29 (4.9)	8 (1.3)	8 (10.7)	2 (2.7)	1
PMS2	16 (2.7)	4 (0.7)	6 (8)	1 (1.3)	1
MLH1	24 (4)	13 (2.2)	9 (12)	2 (2.7)	0.13
POLE	50 (8.4)	11 (1.9)	17 (22.7)	2 (2.7)	0.48
BRCA1	19 (3.2)	5 (0.8)	5 (6.7)	2 (2.7)	0.6
BRCA2	69 (11.6)	18 (3)	21 (28)	8 (10.7)	0.28
ATM	107 (18)	52(8.8)	34 (45.3)	16 (21.3)	1
BRIP1	25 (4.2)	4 (0.7)	7 (9.3)	1 (1.3)	1
CDK12	42 (7.1)	15 (2.5)	10 (13.3)	7 (9.3)	0.07
RNF43	58 (9.8)	38 (6.4)	24 (32)	15 (20)	0.8
LRP1B	190 (32)	44 (7.4)	43 (57.3)	13 (17.3)	0.33
AMER1	72 (12.1)	52 (8.8)	12 (16)	8 (10.7)	0.73
AXIN2	36 (6.1)	22 (3.7)	14 (18.7)	10 (13.3)	0.74
FAT1	99 (16.7)	15 (2.5)	35 (46.7)	4 (5.3)	0.77

**Table 4:** Characteristics of colon cancers with suppressed (z-score relative to normal samples (log RNA Seq V2) below −2) and non-suppressed CDX2 (z-score relative to normal samples (log RNA Seq V2) above 0) from the Sidra-LUMC AC-ICAM cohort. Percentages are shown in parentheses. CMS: Consensus Molecular Subtype, FGA: Fragment Genome Altered, TMB: Tumor Mutation Burden, NA: Not available.

Characteristic	Entire Cohort (n = 348)	CDX2 Suppressed (n = 21)	Non CDX2 Suppressed (n = 219)	*p*
Age				
Mean (±SD)	68.2 ± 11.5	72.3 ± 9.5	68.4 ± 10.7	0.1
>65 years old	233 (67)	15 (71.4)	151 (68.9)	1
≤65 years old	115 (33)	6 (28.6)	68 (31.1)	
Gender				
Male	182 (52.3)	5 (23.8)	123 (56.2)	0.005
Female	166 (47.7)	16 (76.2)	96 (43.8)	
Stage				
I	55 (15.8)	0	46 (21)	
II	122 (35.1)	8 (38.1)	69 (31.5)	1 (Stage I–III versus IV)
III	110 (31.6)	9 (42.9)	65 (29.7)	
IV	61 (17.5)	4 (19)	39 (17.8)	
Primary location				
Cecum-ascending colon–hepatic flexure	158 (45.4)	17 (81)	95 (43.4)	0.001
Transverse colon–splenic flexure-descending colon–sigmoid	190 (54.6)	4 (19)	124 (56.6)	
Consensus Mol Subtype				
CMS1	43 (12.4)	14 (66.7)	5 (2.3)	<0.0001
CMS2	76 (21.8)	0	74 (33.8)	
CMS3	66 (19)	0	52 (23.7)	
CMS4	82 (23.6)	6 (28.6)	40 (18.3)	
Mixed	81 (23.3)	1 (4.8)	48 (21.9)	
FGA				
≤0.08	88 (31.3)	11(68.8)	38 (21)	0.0001
>0.08	193 (68.7)	5 (31.2)	143 (79)	
NA	67	5	38	
TMB				
≤10 mutations/Mb	208 (74)	3 (18.7)	160 (88.4)	0.0001
>10 mutations/Mb	73 (26)	13 (81.3)	21 (11.6)	
NA	67	5	38	

## Data Availability

All data generated in this study are presented in the article and no additional data are available.
